# The Loss of Tafazzin Transacetylase Activity Is Sufficient to Drive Testicular Infertility

**DOI:** 10.3390/jdb12040032

**Published:** 2024-11-26

**Authors:** Paige L. Snider, Elizabeth A. Sierra Potchanant, Catalina Matias, Donna M. Edwards, Jeffrey J. Brault, Simon J. Conway

**Affiliations:** 1 Herman B. Wells Center for Pediatric Research, Indiana University School of Medicine, Indianapolis, IN 46202, USA; 2 Department of Anatomy, Cell Biology and Physiology, Indiana University School of Medicine, Indianapolis, IN 46202, USAjebrault@iu.edu (J.J.B.)

**Keywords:** Barth syndrome, infertility, spermatogenesis, testis, apoptosis, azoospermia, tafazzin

## Abstract

Barth syndrome (BTHS) is a rare, infantile-onset, X-linked mitochondriopathy exhibiting a variable presentation of failure to thrive, growth insufficiency, skeletal myopathy, neutropenia, and heart anomalies due to mitochondrial dysfunction secondary to inherited TAFAZZIN transacetylase mutations. Although not reported in BTHS patients, male infertility is observed in several *Tafazzin* (*Taz*) mouse alleles and in a *Drosophila* mutant. Herein, we examined the male infertility phenotype in a BTHS-patient-derived *D75H* point-mutant knockin mouse (*Taz^PM^*) allele that expresses a mutant protein lacking transacetylase activity. Neonatal and adult *Taz^PM^* testes were hypoplastic, and their epididymis lacked sperm. Histology and biomarker analysis revealed *Taz^PM^* spermatogenesis is arrested prior to sexual maturation due to an inability to undergo meiosis and the generation of haploid spermatids. Moreover, *Taz^PM^* testicular mitochondria were found to be structurally abnormal, and there was an elevation of p53-dependent apoptosis within *Taz^PM^* seminiferous tubules. Immunoblot analysis revealed that *Taz^PM^* gamete genome integrity was compromised, and both histone γ-H2Ax and Nucleoside diphosphate kinase-5 protein expression were absent in juvenile *Taz^PM^* testes when compared to controls. We demonstrate that Taz-mediated transacetylase activity is required within mitochondria for normal spermatogenesis, and its absence results in meiotic arrest. We hypothesize that elevated *Taz^PM^* spermatogonial apoptosis causes azoospermia and complete infertility.

## 1. Introduction

Barth syndrome (BTHS; OMIM# 302060) is an X-linked recessive disorder affecting mainly males and exhibits defects in multiple diverse organ systems [1,2,3]. BTHS is generally characterized by skeletal myopathy, cardiomyopathy, neutropenia, fatigue, and short stature due to inherited *TAFAZZIN (TAZ)* mutations [1,2,3,4,5]. The TAZ transacylase enzyme is crucial for the maturation of cardiolipin (CL), a prominent ubiquitous phospholipid required for normal mitochondrial morphogenesis and function [5,6]. Impaired CL biogenesis causes instability of the inner mitochondrial membrane and destabilization of the respiratory chain complexes, resulting in the disruption of the mitochondria’s ability to produce adenosine triphosphate (ATP) via oxidative phosphorylation (OxPhos) [5,6,7]. More than 200 different *TAZ* single missense, nonsense, or frameshift mutations (rather than large deletions or chromosomal changes) are known to cause BTHS, and most result in the production of mutant proteins with little or no enzymatic function [8]. As with many mitochondriopathies, there is unfortunately no cure for BTHS, and patients often succumb to premature death [2]. Moreover, as the severity of BTHS symptoms varies, genotype–phenotype correlations are incomplete, and the natural course of BTHS through adulthood and its organ-specific roles remain unclear [9,10]. Consequently, a genetic BTHS mouse model is urgently needed to improve mechanistic understanding of this disease’s spectrum and allow for the development of targeted organ-restricted therapies [11].

We generated a patient-tailored BTHS knockin mutant mouse model (*Taz^PM^*) that harbors a patient-derived point mutation (*D75H*) in the critical TAZ acyltransferase domain required to remodel immature monolyso-CL to mature (18:2)4 CL [11]. Significantly, the *Taz^PM^* allele is the only patient-derived mouse model that expresses a stable mutant Taz protein [11]. Notably, surviving *Taz^PM^* male mutants lack sufficient CL and recapitulate the major BTHS phenotypes, including skeletal myopathy, cardiomyopathy, neutropenia, and growth restriction, along with additional neonatal lethality and liver and infertility anomalies [11]. Although male infertility is not usually considered to be a BTHS phenotype [1,2,9], BTHS patients can exhibit delayed puberty, but this was thought to be due to growth deficiency [2]. However, male infertility is also observed in a *Drosophila* model of BTHS [12], as well as in the testes of *Taz* knockout chimeras (*Taz^NEO^*), *Taz* null (*Taz^KO^*), and our *Taz^PM^* knockin mice [13,14], suggesting the potential conserved role of TAZ in male infertility. Moreover, the X chromosome is enriched in genes expressed in early spermatogenesis and male infertility is closely linked with single-X-chromosome dosage deficiencies [15]. Although constant sperm production relies on continually active ATP generation by mitochondria [16] and mammalian testes have been shown to express unique CL species such as (16:0)4 CL and tetrapalmitoyl-CL, a fully saturated species during male reproduction, the testicular function of TAZ remains uncertain [17,18]. Therefore, this study characterizes the developmental pathogenesis of the patient-tailored *Taz^PM^* male knockin mouse mutant infertility phenotype [11] and demonstrates that the loss of Taz acyltransferase activity is sufficient to cause fully penetrant mammalian male infertility. Furthermore, we reveal the spatiotemporal expression pattern of *Taz* within the postnatal testes and the onset of the resultant *Taz^PM^* testis germ cell defects. In addition, we show that *Taz^PM^* mutants exhibit hypoplastic testes, azoospermia and an early p53-associated apoptotic spermatogenesis deficiency.

## 2. Materials and Methods

### 2.1. Mice

Age-matched littermate wildtype (*wt*) and *Tafazzin* (MGI:109626) *D75H* point mutant knockin (*Taz^PM^*) testes were harvested and genotyped as described [11]. The *Taz^PM^* allele was detected using the following specific primers, namely forward-TGGCACAGTAAGAAGCCTCG and reverse-GGGACACACAAAACTTCCAGC, followed by restriction enzyme confirmation (as the *D75H* substitution introduces a new AvaII site, resulting in either positive digest 179 bp + 162 bp mutant bands in heterozygotes or a single uncut 341 bp band in wild-types). Single 8-week and 4-month *Taz^PM^* males (♂) were caged with two 8-week *wt* females (♀) as trios for 6 months, and ♀ were replaced with new 8-week *wt*♀ every 2 months. The numbers of pups, litters, and vaginal plugs in ♀ were counted in each cage.

### 2.2. Histology, Immunohistochemistry and In Situ Hybridization

Following the weighing of the mice, testes were removed and weighed to obtain the ratio of testis weight to body weight using an ultrabalance (Adam Equipment, Columbia, MD, USA). Relative testis sizes were calculated from digital measurements using AxioVision 4.8 software (Zeiss, Oberkochen, Germany) taken using a StemiSVII AxioscopeAPO dissecting scope (Zeiss, Oberkochen, Germany) and seminiferous tubule sizes measured within histological serial sections. Mouse postnatal day (P) 5, 17, 21, 28 and testes and epididymis from 4-month-old mice (n = at least 4/genotype/age) were fixed in 4% paraformaldehyde overnight, dehydrated, embedded in paraffin, and sectioned at 5 and 10 μm. Serial sections (at least 4/slide) were either stained with hematoxylin and eosin or Oil red-O for histological analysis, and used for immunohistochemistry or in situ hybridization analysis of molecular biomarkers, as described [19,20]. Antibodies against Hook1 (1:400 Atlas Antibodies, Stockholm, Sweden) and Dazl (1:400 Abcam, Cambridge, UK) were used to assess spermatogenesis, and phospho-Histone H3 primary antibody (1:500, MilliporeSigma, Temecula, CA, USA) used to measure proliferation, and apoptosis was evaluated using the ApopTag In Situ Detection Kit (MillporeSigma, Temecula, CA, USA) according to the manufacturer. DAB/hydrogen peroxide was used to visualize immunosignals, and antibody diluent (Vectorstain, Newark, CA, USA), without primary antibodies, was used for negative controls. The mean ± SD number of cells labeled by pHH3 (proliferation index) or TUNEL (apoptotic index) were determined per 1000 cells counted in 6 separate fields in 5 mm testis sections (n = 4 separate testis/stage/genotype. Sense and anti-sense non-radioactive *Catsper1* (MGI:2179947), *Catsper4* (MGI:3043288), *Nme5* (MGI:1922783) and *Cracd* (MGI:2444817) cDNA probes were transcribed for hybridization. Two partially overlapping *Taz* (MGI:109626) cDNAs probes were generated from 218 to 801 bp (583 bp) and from 789 to 1354 bp (565 bp) regions, and both antisense probes produced similar expression patterns in consecutive 10 μm serial sections. Sense probes were negative.

### 2.3. Sperm and Seminiferous Tubule Analysis

The 4-month cauda epididymal spermatozoa were dispersed in phosphate-buffered saline and placed into a hemocytometer; the sperm number was counted per sample, as described [21]. Sperm were collected via 300 g centrifugation, air-dried onto slides and stained with Mito-tracker and DAPI (both ThermoFisher Scientific, Waltham, MA, USA), as well as Actin (Sigma-Aldrich, St. Louis, MO, USA) to analyze spermatogonial morphology. From randomly chosen *wt* and *Taz^PM^* sections (n = 20/genotype/stage), the seminiferous tubule diameter was measured across the minor and major axes, and the mean diameter was obtained.

### 2.4. Ultrastructural Analysis

For transmission electron microscopy, P28 testes were isolated and fixed in 2% paraformaldehyde and 2% glutaraldehyde fixative in 0.1 M cacodylate buffer (pH7.2) overnight, processed, and imaged on a Phillips400 microscope (via IU EM Core) as described [11]. Mitochondrial morphology was performed blinded to genotype and analyzed using ImageJ (version 1.54j).

### 2.5. Nucleotide Measurements

Micro-dissected testes were immediately freeze-clamped in liquid nitrogen and stored at −80 °C until analysis. Frozen samples were homogenized in ice-cold 80% methanol/20% water using Kontes glass tissue grinders to remove protein. Ultra-performance liquid chromatography (UPLC) analyses were used to measure adenine nucleotides in the extracts with UV-Vis and mass spectrometry (MS) simultaneously, as described [22].

### 2.6. Western Analysis

For Western blot analysis, P28 testes were isolated from *Taz^PM^*♂ and *wt*♂ mice (n = 6/genotype) were homogenized in RIPA protein lysis buffer (Sigma-Aldrich, St. Louis, MO, USA) and resultant protein concentrations normalized as described [11,23]. Following blocking in Blotting-Grade Blocker (Bio-Rad, Hercules, CA, USA), blots were probed with the following antibodies: p53 phospho-S392 #33889, Hsp70 #5439, gamma H2AX #81299, Nme5 #231631, Sirt1 #12193 (Abcam, Cambridge, UK), total p53 #sc-71820, Taz #sc-365810 (Santa Cruz, Dallas, TX, USA), GAPDH #G8795 (Sigma-Aldrich, St. Louis, MO, USA), and Vdac #PA1-954A (ThermoFisher, Waltham, MA, USA). Immunoreactive protein band signals were detected and verified as described [11,23]. X-ray films of varying exposures were scanned for each antibody, and densitometric signal intensity was quantified (n = 6 of each genotype/antibody). Statistical analysis was performed via Prism software version 5.02 (GraphPad Software, San Diego, CA, USA).

### 2.7. qPCR

Total RNA was isolated from the testes of *wt*♂ newborn, P5, P10, P17, P21, P28 and 4-month-old mice (n = 4 pairs/age) and cDNA as described [11]. Triplicate samples were amplified in duplicate with *Taz* and housekeeping *PPia* mouse-specific PCR primers as described [11,19]. Cycle number as a quantitative 2^−ΔΔCT^ estimate of the initial template concentration in a sample was quantified from at least n = 4 duplicate samples and normalized to *PPia* (the most stable reference gene expressed during mouse testis development [24]). Statistical analysis was performed via Prism software version 5.02 (GraphPad Software).

### 2.8. Statistical Analysis

Data are presented as mean ± SD from independent experiments with different biological samples per group. Unpaired one- and two-tailed *t*-tests were used for statical comparisons between 2 groups, with significance set at *p* < 0.05, *p* < 0.01, *p* < 0.001 and *p* < 0.0001.

## 3. Results

### 3.1. Postnatal Taz mRNA Expression in the Testis

**Figure 1:**
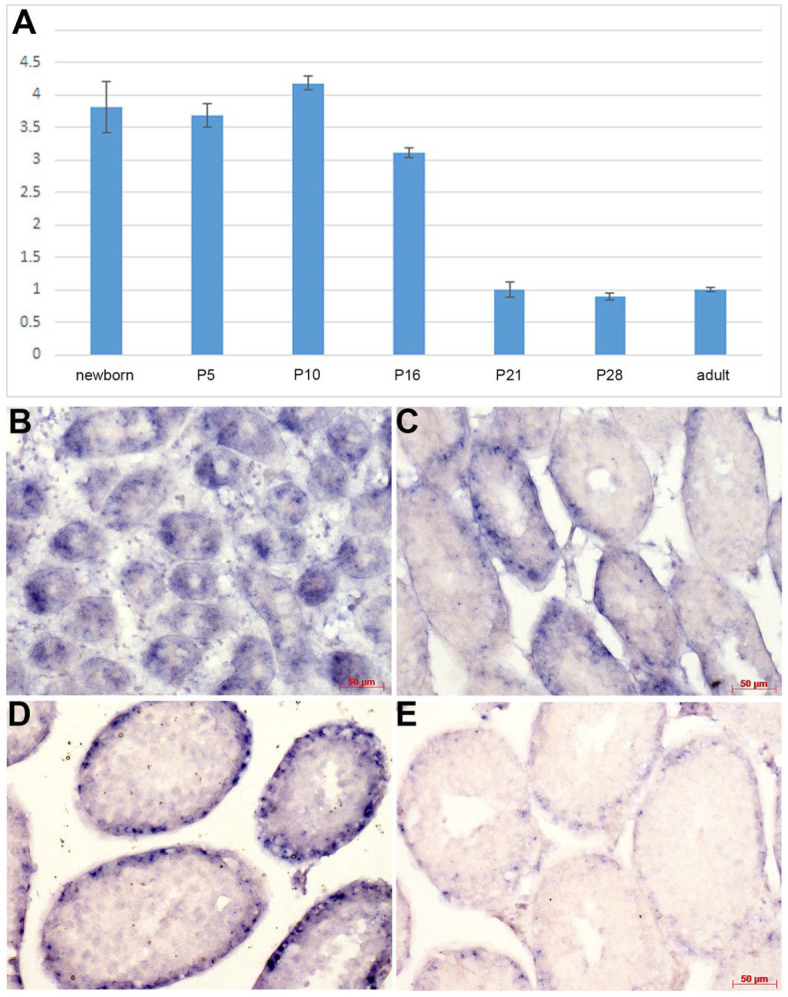
*Tafazzin* mRNA expression during postnatal *wt* mouse testis growth. (**A**) Quantitative triplicate PCR revealed *Taz* levels are robust in newborn, P5, P10 and P16 testes (averaging ~3.67-fold), but is expressed at basal levels in P21, P28 and adult (4-month) testes. *Taz* expression was compared to housekeeping gene *Ppia*. qPCR data are presented as a logarithmic plot of relative expression, where a value of 1 indicates no difference in 4-month-old adult testes and values < 1 indicate reduced and >1 indicate increased expression. The Y-axis is the relative fold difference, and error bars represent SD. (**B**–**E**) Non-radioactive in situ hybridization detection of *Taz* (purple precipitate) in staged postnatal mouse testis sections revealed punctate robust expression within nascent seminiferous tubules at P5 (**B**), P16 (**C**) and P21 (**D**), but only low-level expression in P28 seminiferous tubules (**E**). *Taz* signal was only observed when sections were hybridized with the *Taz* anti-sense probe, confirming signal specificity. Serial sections were examined for comparable spatiotemporal patterns in at least three consecutive serial sections/stage. Scale bars (**B**–**E**) = 50 μm.

Although *Taz* deficiency in *Drosophila* as well as in both chimeric *Taz^NEO^* and knockout *Taz^KO^* mutant mice can cause male sterility [12,13,14], it remains unclear where and when *TAZ* is expressed in mammalian testes. To accurately recognize the effect of our point-mutant knockin (*Taz^PM^*) and infer its function, we examined *Taz* mRNA expression levels and distribution in *wt* mice testes. qPCR analysis revealed *Taz* expression is present in testes at significant levels from newborn to postnatal (P) 16 stages, but is reduced in P21 to 4-month-old adults (Figure 1A). In situ hybridization confirmed localized robust *Taz* expression in P5 testes (when the spermatogonia first begin to differentiate), specifically within all the immature spermatogonia located in the wall of the seminiferous tubules (Figure 1B). At P16 and P21 (when the majority of the seminiferous tubules exhibit spermatocytes as the most advanced germ cell type but some tubules exhibit some round spermatids), restricted *Taz* expression continues robustly in testis tubules in basally located cells, many of which are spermatogonia, and some spermatocytes (Figure 1C,D). However, only weak *Taz* expression was observed in the basal layer of the P28 tubules (when elongating spermatids appear; Figure 1E) and in adult testes. Thus, *Taz* mRNA is present in both the immature postnatal and adult mice testes, with the highest level of expression occurring during the first wave of meiosis and spermatogenesis, continuing to be expressed at low levels in P28 and adult testes.

### 3.2. Taz^PM^ Testes Are Hypoplastic, Causing Infertility

**Figure 2:**
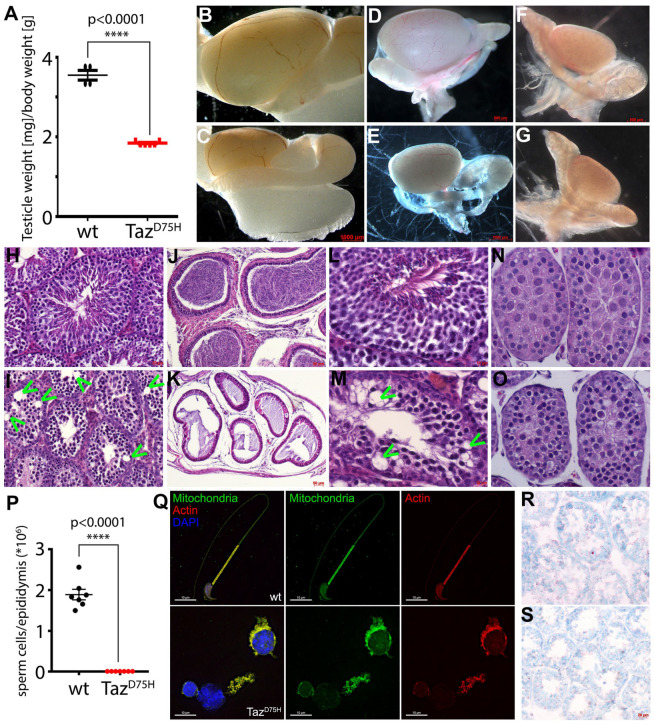
Characterization of *Taz^PM^* testis phenotype. (**A**) Adult 4-month testis/body weight ratio in *wt* (black, n = 4) verses *Taz^PM^* knockin (red, n = 6) mice. (**B**–**G**) Representative images of whole *wt* (**B**,**D**,**F**) and *Taz^PM^* (**C**,**E**,**G**) 4-month (**B**,**C**), P17 (**D**,**E**) and P5 (**F**,**G**) littermate testes. (**H**–**O**) Hematoxylin and eosin staining of *wt* (**H**,**J**,**L**,**N**) and *Taz^PM^* (**I**,**K**,**M**,**O**) testes (**H**,**I**) and epididymis (**J**,**K**) from 4-month-old mice, as well as P28 (**L**,**M**) and P17 (**N**,**O**) testis sections. (**P**,**Q**) Analysis of 4-month epidydimal *wt* and *Taz^PM^* content numbers ((**P**), n = 7/genotype) and resultant staining of sperm head nuclei DNA chromatin (blue DAPI-positive), mitochondria-rich midpiece (green MitoTracker-positive) and tail (red actin-positive) within contents. (**R**,**S**) Oil red-O staining of lipid deposition in P28 *wt* (**R**) and *Taz^PM^* (**S**) testes. Scale bars (**B**,**C**,**E**) = 1 mm; (**D**,**F**,**G**) = 500 μm; (**H**,**I**,**K**,**R**,**S**) = 20 μm; (**J**,**K**) = 50 μm; (**L**,**M**,**N**,**O**,**Q**) = 10 μm. Statistical significance set at **** *p* < 0.0001.

We previously reported that surviving hemizygous knockin males (*Y/Taz^PM^*♂) were infertile, whereas heterozygous knockin females (*Taz^wt^/Taz^PM^*♀) were fertile [11]. To determine whether *Taz^PM^*♂ infertility was primarily due to reproduction defects or secondarily due to the observed *Taz^PM^*♂ growth delay and/or progressive cardiomyopathy phenotype/skeletal muscle hypoplasia [11], we tested whether young 8-week-old (when mice usually reach sexual maturity) and larger more mature 4-month-old *Taz^PM^*♂ are equally infertile. To test fertility, we crossed six 8-week and six 4-month *Taz^PM^*♂ with wildtype (*wt*) ♀ mice and counted the litter and/or litter size born within a 6-month period. Significantly, no litters resulted for either age (n = 0/6 per age), despite the presence of copulatory plugs, suggesting a critical fertility alteration. These data indicate that growth restriction, potential immaturity and/or inactivity are not causes of the observed *Taz^PM^*♂ infertility. Next, we examined the reproductive system and found that adult *Taz^PM^* paired-testis weights were significantly smaller (Figure 2A) as the testis/body weight ratio of adult *Taz^PM^* knockin was reduced ~50% compared to *wt* littermates (Figure 2B,C). Temporal analysis revealed that, grossly, *Taz^PM^* testes start to exhibit size limitation beginning around P5, which results in a ~30% reduction by P17 in *Taz^PM^* mice (Figure 2D–G). Moreover, the epididymis of *Taz^PM^* mice also exhibited similar growth restrictions (Figure 2K).

Histology revealed adult *Taz^PM^* seminiferous tubules and epididymis have reduced diameters (adult *wt* 176 ± 15 μm vs. *Taz^PM^* 81 ± 9 μm, *p* = 0.01) and appear to lack mature sperm, with the usual *wt* spermatogonial layered architecture of progressively differentiating spermatocytes being perturbed in *Taz^PM^*, with fewer cells present and frequent vacuole interruptions, as well as no spermatozoa in the lumen (Figure 2H,I). However, *Taz^PM^* interstitial Leydig cells and myoepithelial/epithelial cells of the duct system appear grossly unaffected. Analysis of adult epidydimal contents revealed the presence of motile structurally normal sperm in *wt* mice, while only a few sloughed off immotile immature spermatogonia were observed in *Taz^PM^* (Figure 2J,K,P,Q). Further analysis of germ cells in the testis sections at earlier juvenile stages of spermatogenesis revealed that P28 and P17 *Taz^PM^* testes exhibit morphological defects, including smaller seminiferous tubule diameters (P28 *wt* 118 ± 11 μm vs. *Taz^PM^* 79 ± 17 μm, *p* = 0.01), disruption of sequential layers along with a buildup of immature spermatogonia, vacuoles (indicated via arrowheads) and only a few large pachytene spermatocytes but no diplotene spermatocytes compared to *wt* littermates (Figure 2L–O). Additionally, *Taz^PM^* seminiferous tubules contain cells with cytoplasmic and nuclear condensation that may be apoptotic (Figure 2O). As the spermatocyte pachytene stage starts ~P10 [25], we examined P5 testes to assess the earlier onset of spermatogenesis [26]. Histology did not reveal any gross morphological nor lumen size differences, suggesting that the lack of *Taz^PM^*♂ acyltransferase activity is required postnatally from P6+ onwards. To determine whether the *Taz^PM^* mutation affects testicular lipid levels as observed in *Taz^PM^*♂ hearts and livers [11], we examined lipid deposition. Unlike either the excess lipid deposition in *Taz^PM^*♂ cardiomyocytes or lipid absence in *Taz^PM^*♂ livers, Oil Red-O staining revealed normal lipid deposition in P28 *Taz^PM^* testes (Figure 2R,S). Combined, this suggests compromised *Taz^PM^* spermatogenesis during early meiosis.

**Figure 3:**
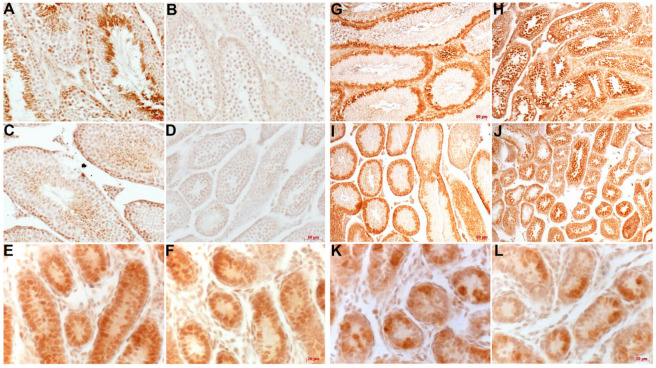
Hook1 and Dazl biomarker analysis. (**A**–**F**) Immunostaining using Hook1 antibody (signal is brown DAB precipitate) expression in 4-month (**A**,**B**), P17 (**C**,**D**) and P5 (**E**,**F**) *wt* (**A**,**C**,**E**) and *Taz^PM^* (**B**,**D**,**F**) testis sections. (**G**–**L**) Immunostaining using Dazl antibody (signal is brown DAB precipitate) expression in 4-month (**G**,**H**), P17 (**I**,**J**) and P5 (**K**,**L**) *wt* (**G**,**I**,**K**) and *Taz^PM^* (**H**,**J**,**L**) testis sections. Scale bars (**A**,**B**,**E**,**F**,**K**,**L**) = 20 μm; (**C**,**D**,**G**,**H**,**I**,**J**) = 50 μm.

To further identify the cell types that disappeared, we employed biomarker immunostaining on seminiferous tubule sections of P5, P17 and adult mice. Deleted in azoospermia-like (Dazl) is a RNA-binding protein required for male gametogenesis that is expressed in spermatogonia and early/late spermatocytes [27], whilst Hook microtubule tethering protein 1 (Hook1) is a microtubule tethering protein that facilitates actin and microtubule binding activity and is predominantly expressed in spermatocytes/haploid spermatids [28]. Significantly, Hook1 is no longer present in P17 and adult *Taz^PM^* tubules, indicating an absence of *Taz^PM^* round spermatids and differentiated spermatozoa (Figure 3A–D). Moreover, although Dazl is still expressed in P17 and adult *Taz^PM^* tubules, the typical *wt* basal restriction of Dazl expression within immature spermatogonia/spermatocytes is absent, and instead immature Dazl-positive cells extend throughout the mutant tubules (Figure 3G–J). In agreement with the P5 histological data, both Dazl and Hook1 proteins are normally expressed in both P5 *Taz^PM^* and *wt* seminiferous tubules spermatogonia (Figure 3E,F,K,L). Combined, these data demonstrate that atrophying *Taz^PM^* testes are infertile, with early spermatogenesis arrest prior to sexual maturation due to an inability to generate haploid spermatids.

### 3.3. Molecular Analysis of Taz^PM^ Testis Anomalies

**Figure 4:**
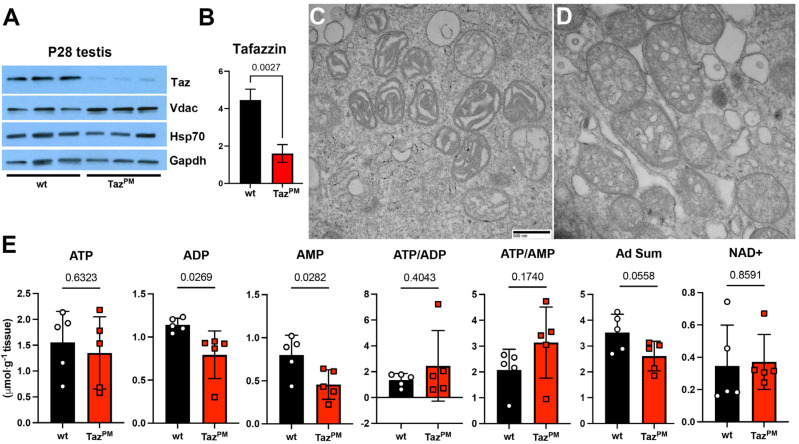
Molecular analysis of *Taz^PM^* testis phenotype. (**A**,**B**) Western evaluation of Taz and Vdac protein levels in triplicate *wt* and *Taz^PM^* P28 testes ((**A**), n = 3 per genotype). Both mitochondria Hsp70 and housekeeping Gapdh were used as independent loading controls, with Taz; Gapdh quantification and statistical analysis (via two-tailed t-test) shown (**B**). The Y-axis is the relative fold difference. (**C**,**D**) Representative electron microscopy images showing P28 *wt* normal (**C**) and *Taz^PM^* swollen/abnormal (**D**) mitochondrial morphology (n = 4/genotype). (**E**) Ultra-performance liquid chromatography analysis (n = 5/genotype) revealed that ATP, ADP, AMP, and NAD^+^ levels as well as ATP/ADP and ATP/AMP ratios remain unchanged, but total adenine nucleotides (Ad Sum) are reduced (*p* = 0.05) in *Taz^PM^* (red) verses *wt* (black) testes (n = 5/genotype). Scale bars (**C**,**D**) = 500 nm.

As we previously demonstrated, *Taz^PM^*♂ cardiac ventricles, gastrocnemius skeletal muscles and livers express a mutant Taz^PM^ protein at equivalent levels to *wt*♂ [11]. We used Western blotting to measure Taz protein expression in testes. Significantly, P28 *Taz^PM^* testes express the mutant Taz^PM^ protein at significantly lower levels (*p* = 0.0027) when compared with *wt* littermate testes (Figure 4A,B), suggesting that *Taz^PM^* testis abnormalities may involve a different pathogenic phenotype compared to *Taz^PM^*♂ striated muscles and livers. To assess whether decreased Taz^PM^ is due to there being fewer mitochondria, we measured voltage-dependent anion channels (Vdac) levels, as it is a major component of the outer mitochondrial membrane and a useful mitochondrial loading control [29]. As both Vdac (*p* = 0.249) and mitochondrial Hsp70 levels are not significantly altered, this indicates that *Taz^PM^*♂ mitochondria are present at similar levels to *wt* (Figure 4A). As mitochondrial defects are predominant in BTHS, and TAZ itself impacts many aspects of mitochondrial structure function [4,6], we examined how the loss of *Taz^PM^*♂ transacylase activity may affect mitochondrial morphogenesis and energy production, as ATP synthesis is one of the mitochondria’s primary functions [30], and oxidative phosphorylation (OxPhos) is required for male fertility [16,31]. Mitochondrial ultrastructure was examined via transmission electron microscopy, and all P28 *Taz^PM^* testis mitochondria are structurally abnormal when compared to *wt*♂ littermates (Figure 4C,D). *Taz^PM^*♂ mitochondria exhibit an enlarged honeycomb-like phenotype with a markedly lower number of cristae and a lack of contiguous intermembrane/intracristae spaces compared to *wt*♂ littermates that contain orthodox state cristae extending across the entire body of the organelle, affording an extensive surface area. However, despite these structural anomalies, *Taz^PM^* testis ATP and NAD^+^ levels were unperturbed, and there is no decrease in ATP/ADP or ATP/AMP ratios (Figure 4E), suggesting that *Taz^PM^* testes are not energetically adverse. Interestingly, ADP (*p* = 0.027) and AMP (*p* = 0.028) levels and the total adenine nucleotides (ATP + ADP + AMP, *p* = 0.05) are all significantly lower in *Taz^PM^* than in *wt* littermate testes (Figure 4E), implying an accelerated nucleotide degradation [32] or a change in cell type [33] in abnormal *Taz^PM^* testes.

### 3.4. Spermatogenic Biomarker Analysis

**Figure 5:**
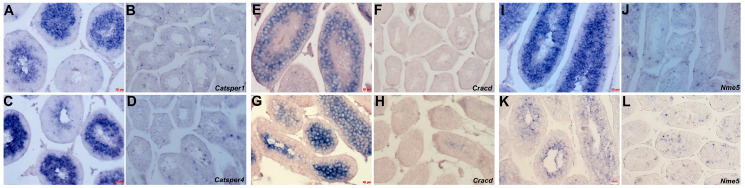
Spermatogenic failure in *Taz^PM^* testes. (**A**–**D**) Non-radioactive in situ hybridization detection of *Catsper1* (**A**,**B**) and *Catsper4* (**C**,**D**) mRNA in immature P28 *wt* (**A**,**C**) and *Taz^PM^* (**B**,**D**) testis sections. (**E**–**H**) *Cracd* mRNA in P28 (**E**,**F**) and P17 (**G**,**H**) *wt* (**E**,**G**) and *Taz^PM^* (**F**,**H**) testis sections. (**I**–**L**) *Nme5* mRNA in P28 (**I**,**J**) and P17 (**K**,**L**) *wt* (**I**,**K**) and *Taz^PM^* (**J**,**L**) testis sections. Scale bars (**A**–**J**) = 20 μm; (**K**,**L**) = 10 μm.

To gain a more detailed understanding of the progressive effects of *Taz^PM^* mutation within testes, we examined the expression of several spermatogenic effectors. In agreement with our histology and Dazl/Hook1 biomarker data (see Figure 2 and Figure 3), the expression of *Catsper1* and *Catsper4* mRNAs is mostly abolished in adult [34,35] and P28 *Taz^PM^* testes (Figure 5A–D). As these cation channels of sperm play key roles in meiosis during spermatogenesis and sperm function [34,35], these data reinforce the conclusion that meiosis is directly compromised in *Taz^PM^* testes. Moreover, spermatogenesis marker *Cracd* mRNA expression is similarly absent in adult, P28 (Figure 5F) and P17 (Figure 5H) *Taz^PM^* testes. This further suggests that early meiosis during spermatogenesis [19] is affected in *Taz^PM^* testes, even prior to an overt phenotype being evident. As *Non-metastatic cells-5* (*Nme5*) is thought to be required for mitotic and meiotic differentiation of spermatozoa [36,37], we examined its expression. Significantly, *Nme5* mRNA is also downregulated in both P28 and P17 *Taz^PM^* testes (Figure 5J,L). Combined, these results suggest that the *Taz^PM^* phenotype may be due to either compromised spermatogenic differentiation prior to meiosis or meiotic arrest itself.

**Figure 6:**
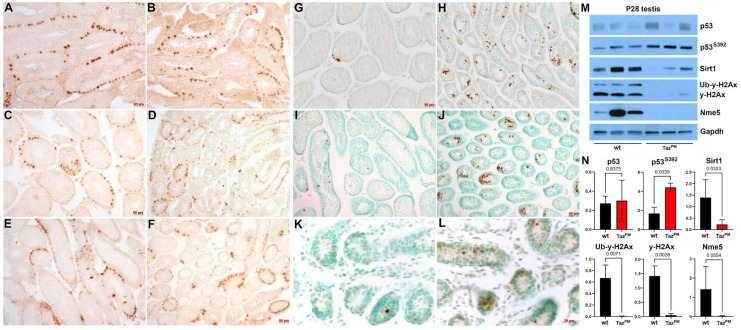
*Taz^PM^* testes exhibit persistent p53-associated apoptosis. (**A**–**F**) Immunohistological detection of phosphohistone H3 cell proliferation marker (punctate brown DAD-positivity) in 4-month (**A**,**B**), P28 (**C**,**D**) and P17 (**E**,**F**) *wt* (**A**,**C**,**E**) and *Taz^PM^* (**B**,**D**,**F**) testis sections. (**G**–**L**) TUNEL apoptosis marker expression (punctate brown DAD-positivity) in methyl green counterstained P28 (**G**,**H**), P17 (**I**,**J**) and P5 (**K**,**L**) *wt* (**G**,**I**,**K**) and *Taz^PM^* (**H**,**J**,**L**) testis sections. (**M**,**N**). Western analysis of total p53, phospho p53^S392^, Sirt1, γ-H2Ax (both upper ubiquitinated and lower phospho γ-H2Ax isoforms) and Nme5 protein levels was performed in triplicate for *wt* and *Taz^PM^* P28 testes ((**M**), n = 3 per genotype). Housekeeping Gapdh was used as the loading control, with quantification and statistical analysis (via two-tailed *t*-tests for all except Sirt1/Nme5 which used one-tailed t-tests due to *wt* variability) shown (**N**). Scale bars (**A**–**J**) = 50 μm; (**K**,**L**) = 20 μm.

Given the hypoplastic appearance of *Taz^PM^* testes, we examined both cell proliferation and apoptosis to determine if they may play a role in the observed growth restriction (see Figure 2). The phosphohistone H3 marker revealed that cell proliferation indexes were unaffected in adult, P28 and P17 (Figure 6A–F) *Taz^PM^* testes. However, terminal deoxynucleotidyl transferase dUTP nick end labeling (TUNEL) revealed that the cellular apoptotic indexes were significantly increased in adult, P28 (Figure 6H) and P17 (Figure 6J) *Taz^PM^* testes within the basal layer of the seminiferous tubules, when compared to typical *wt* levels that occur during normal spermatogenesis [38]. Indeed, modest increased TUNEL labeling is also present in P5 (Figure 6L) *Taz^PM^* tubules, suggesting that apoptosis is activated when spermatogonia first begin to differentiate. Given these data and the histological presence of seminiferous tubule vacuoles and cells containing cytoplasmic and nuclear condensation (see Figure 2), this implies that apoptotic cell dropout is responsible for the reduced testis size and the absence of mature spermatogonia, and there is persistent *Taz^PM^* apoptosis that continues into adult life.

As transformation-related protein 53 (p53) transcription factor is highly expressed in the testes, can regulate the apoptotic process in spermatocytes and spermatogonia, and is associated with male infertility [39,40], we examined its protein levels. Although we previously demonstrated that total p53 and several p53 effectors are mis-expressed in *Taz^PM^*♂ hearts [11], total p53 levels were unaltered in P28 *Taz^PM^* testes (*p* = 0.8375). Moreover, despite p53 phosphorylation being unaffected in juvenile and abolished in adult mutant hearts [11], serine 392 phosphorylation of p53 [41] was increased significantly (*p* = 0.0039) in *Taz^PM^* testes (Figure 6M,N). However, protein levels of both p53-interacting Sirt1 (which deacetylates p53 reducing its protein stability and transcriptional activity [42]) and phospho S139 histone γ-H2Ax (a DNA damage marker and repair effector [43,44]) that increase with spermatogonia differentiation are significantly downregulated (*p* = 0.03 and *p* = 0.007/0.002, respectively) in P28 *Taz^PM^* testes (Figure 6M). Finally, in addition to the observed spermatogonia and early spermatocyte *Nme5* mRNA downregulation (see Figure 5), we found that Nme5 [36,37] protein levels are also significantly (*p* = 0.05) reduced in P28 *Taz^PM^* testes (Figure 6M). Collectively, these biomarkers suggest that *Taz^PM^*♂ infertility may occur as a secondary effect due to elevated p53-associated apoptosis, and there is an inability to undergo normal meiosis and/or resolve DNA damage and repair it in mutant *Taz*-expressing spermatogonia.

## 4. Discussion

We generated the first patient-tailored BTHS knockin mutant mouse model (*Taz^PM^*) that lacks acyltransferase activity but expresses a stable mutant protein, resulting in a buildup of immature MLCL relative to CL [11]. Our objective was to examine why *Taz^PM^*♂ mice are infertile, to determine when and where *Taz* mRNA is initially expressed in postnatal testes, and to uncover the underlying pathogenic mechanism. Significantly, *Taz* is expressed in a restricted manner within *wt* P5 seminiferous tubules onwards, and we observed resultant *Taz^PM^* testis growth restriction from P5 onwards. Histology and spermatogenic biomarker analysis revealed that *Taz^PM^* seminiferous tubules contained an abnormal accumulation of spermatogonia, that the P17 first wave of *Taz^PM^* spermatogenesis was perturbed (shown via expansion of Dazl and the absence of Hook1, *Cracd* and Nme5), and that undersized *Taz^PM^* testes and epididymis lack any haploid spermatids/sperm. Significantly, these mutant juvenile testes appear to completely lack differentiated spermatocytes/round haploid spermatids (shown by the absence of Hook1, *Catsper1,4*) and thus exhibit compromised spermatogenic differentiation prior to meiosis or meiotic arrest itself. Moreover, there was a significant increase in the number of TUNEL-positive cells in *Taz^PM^* testes from P5 onwards, particularly within the mutant seminiferous tubule atypical spermatogonia. These results suggest that the absence of Taz function hinders spermatogenic differentiation, or causes it to progress incorrectly. Indeed, during regular mammalian spermatogenesis, more than 75% of the developing spermatogenic cells can undergo apoptosis before maturation to remove excess and/or damaged germ cells [45], and we suggest that the lack of spermatogonial Taz acyltransferase activity is sufficient to cause elevated activation of apoptosis, which results in the observed fully penetrant *Taz^PM^* azoospermia and infertility.

Intriguingly, the *Taz^PM^* testis phenotype appears to be unique across tissues within the *Taz^PM^* model. As we previously demonstrated, *Taz^PM^*♂ hearts do not undergo apoptosis despite being growth-restricted; moreover, adult *Taz^PM^*♂ hearts contain reduced ATP levels and ATP/ADP ratios, adult *Taz^PM^*♂ hearts and livers both exhibit dysregulated abnormal lipid deposition, *Taz^PM^*♂ hearts exhibit abnormal total p53 levels, and the mutant Taz^PM^ protein is expressed at equivalent levels in mutant hearts, skeletal muscle, and livers when compared to *wt*♂ littermates [11]. As in situ data revealed that *Taz* is robustly expressed mainly within P5–P16 seminiferous tubules, and that apoptosis is elevated in mutant testes from P5 onwards, this suggests that the reduced levels of mutant Taz^PM^ protein in *Taz^PM^* testes may be a consequence of *Taz*-expressing cell apoptotic removal. Similarly, in contrast to mutant hearts, there was no decrease in ATP/ADP or ATP/AMP levels in *Taz^PM^* testes, suggesting that they are not energetically worse [46]. However, there was a significant change in total nucleotides within *Taz^PM^* testes that may well be due to an apoptotic-mediated alteration in cell type (mutant spermatogonia preferentially undergo apoptosis), as different cells can have variable amounts of basal ATP [33]. Finally, the finding that only *Taz^PM^* testes exhibit abnormal apoptosis is most likely due to a consequence of the unique existence of germ cell meiosis during the production of haploid gametes [47], which is not present in somatic cell lineages. During meiosis, many organelles including the mitochondria undergo dramatic structural and functional remodeling to be inherited in gametes, including adaptations to increased energetic needs, differential mitochondrial protein expression, mitochondrial fusion/translocation, and a metabolic shift from glycolysis to OxPhos [29,31,46,48]. Significantly, although testicular mutant mitochondria are present at a similar density as *wt*, the *Taz^PM^* mitochondria are dysmorphic and have a reduced cristae and surface area, suggesting that OxPhos may be perturbed [49]. Further studies will be necessary to determine whether the electron transport chain complex levels and clustering are directly affected. Moreover, there are also extensive epigenetic modifications that preserve haploid gamete genome integrity, induce and repair programmed DNA double-strand breaks, inhibit transposable elements, and reprogram chromatin to support totipotency in the fertilized zygote [13,50]. Our data suggest that mutant genome integrity may be compromised, as Nme5-mediated synthesis of nucleoside triphosphates for DNA/RNA synthesis [36,37] and histone γ-H2Ax-mediated programmed DNA double-strand break repair [43,44] biomarkers are both absent in juvenile P28 *Taz^PM^* testes. Moreover, whilst p53-associated Sirt1 is absent (*Sirt1^KO^* testis gametes exhibit increased apoptosis [51]), p53^S392^ phosphorylation is upregulated in P28 *Taz^PM^* testes, indicating the presence of dysregulated p53-dependent apoptosis in mutant testes. Indeed, γ-H2Ax disappears coincidentally with apoptosis progression [52] and is required to maintain genome stability in cooperation with p53 [53]. Combined, these data suggest that *Taz^PM^* gametes either undergo apoptosis before double-strand break induction, or that the double-strand break is never formed. Future studies will be required to determine both the extent of mitochondrial complex alterations and the direct causes of early meiotic arrest in *Taz^PM^* gametes.

Although p53 has multiple pathological and physiological roles, it is known to play a key role during spermatogenesis, particularly in protecting germline integrity. Specifically, p53 can control spermatocyte meiotic progression and crossover formation [54], repair meiotic double-stranded breaks [55], and drive DNA-damage-induced apoptosis of germ cells [39,40,56]. Thus, upregulation of the testicular p53 pathway is necessary to correct and/or eliminate DNA-damaged gametes that, if unchecked, would lead to abnormal differentiation or irreversible arrest. Our finding that p53^S392^ phosphorylation is upregulated in juvenile *Taz^PM^* testes, coincident with the observed meiotic arrest, suggests that the lack of Taz function itself results in elevated DNA damage in mutant gametes. Supporting this, we observed that both Sirt1 and Nme5 levels were significantly diminished, suggesting that a failure to eliminate reactive oxygen species may enhance mutant DNA damage. As p53 can also be activated in response to nucleotide depletion and/or nucleotide imbalance causing replication defects [56,57], the combined *Taz^PM^* testicular AMP/ADP imbalance and lack of protective Sirt1/Nme5/γ-H2Ax may work together to trigger the robust pro-apoptotic p53 response. Whether this is due to reduced levels of Taz^PM^ proteins or the presence of the *D75H* point mutation itself, and whether p53 can be activated in *Taz^KO^* testes, remains unclear. The absence of the γ-H2Ax signal in *Taz^PM^* testes suggests that apoptosis is induced prior to the formation of programmed double-stranded DNA breaks. Interestingly, high-percentage *Taz^Neo^* chimeras [13] that likely lack the Taz protein exhibit increased γ-H2Ax expression, suggesting that either different mutations exhibit diverse phenotype–genotype correlations and/or Taz^PM^ itself may drive p53 activation. Indeed, it remains unknown as to what role Taz^PM^-mediated p53-dependent apoptosis may play within the diverse and variable BTHS phenotypes observed, as p53 can cooperate with Sirt’s to regulate CL biosynthesis [11], and disrupted CL biosynthesis can prevent p53 mitochondrial translocation and protection of the mitochondrial genome [58].

Even if BTHS has not been thought to be closely associated with male infertility, BTHS patients can exhibit delayed puberty [2]. However, given there are only a small number of BTHS patients, and the difficulty in obtaining tissue samples, allied to the findings that stillbirths and prenatal loss are associated with BTHS [2,59] and the X-chromosomal location of the *TAZ* gene, it remains unclear whether BTHS patients have compromised fertility. Nevertheless, the consistent findings of fully penetrant male infertility in several divergent *Taz* mouse alleles [10,11,13,14], as well as in a *Drosophila* mutant [12], suggest that further BTHS fertility studies and elucidation of p53′s testicular role are warranted.

## Data Availability

All data supporting the findings can be found within the manuscript.
